# The Prevention of Tuberculosis

**Published:** 1903-03-21

**Authors:** 


### The Prevention of Tuberculosis.

Ever since Professor Koch's epoch-making discovery of the tubercle bacillus in 1882 the hope has been gaining ground that one day not too far distant tuberculosis may cease to be the most destructive of the diseases to which mankind is liable, if, indeed, it is too much to expect that the affection may be eventually eliminated altogether from the civilised races. But there are those among us who can but feel some disappointment when they consider that, although for 21 years scientific observers in all parts of the world have been expending an enormous amount of labour and time in thoroughly investigating the nature and characters of the tubercle bacillus, and that they have so far succeeded in their researches that at the present moment our knowledge of this parasite is probably more complete than that of any other similar organism, yet, in spite of all this, little, if anything, has so far been achieved as a result towards diminishing the prevalence of tuberculosis. The annual mortality from pulmonary consumption in England has been steadily declining since 1838, but the rate of decline has shown no increase whatever since the date of Professor Koch's discovery. The fall of the annual death rate from consumption which has been in progress since 1838, before which date no statistics are available, has been almost without a doubt due to the improved conditions under which the poorer classes have lived and to the increasing regard which has been shown by all ranks to matters of hygiene and sanitation. Doubtless much good has been recently done, mainly in the direction of the establishment of sanatoria, and in the encouragement of a more widespread knowledge of the subject, by the National Association for the Prevention of Consumption, whose fourth annual meeting was held on March 17th. But these measures are not sufficient. What is wanted is the provision of some legislative powers which will enable medical practitioners to enforce that degree of disinfection and isolation which is absolutely essential for the prevention of the wholesale dissemination of this infectious disease on the part of those unfortunate individuals who are suffering from advanced phthisis. What sound laws are capable of effecting in the diminution of certain complaints is conclusively shown in the history of small-pox. The fact that pulmonary tuberculosis is a disease which is communicable from man to man cannot for one moment be denied. Moreover, it would seem, that of all such affections to which human beings are liable, phthisis is the one in which infection should be most easily avoided, since there is a good deal of evidence to show that, in order to contract the disease, a particular individual, who is exposed to the infection, must not only have a lower power of resistance at the time, but the exposure itself must either be prolonged or else frequently repeated. Probably the most common cause of the spread of phthisis is the at present unavoidable custom among the poorer classes of sleeping many in one room, possibly with closed windows and little or no ventilation. If one member of such a family contracts consumption the circumstances under which the rest of the family are situated render the danger of infection to other members very considerable.

The objects which legislation should have in view are the following:—(1) Compulsory notification of all cases of tuberculosis; (2) the isolation of every case of advanced phthisis; (3) the enforcing upon the public at large of the fact that consumption is infectious. There should be no difficulty in effecting the first reform; with regard to the second the initial expense and difficulty would doubtless be great, but whether judged from a humanitarian point of view or from the standpoint of the practical economist, the subject makes an equally powerful appeal. It is one of urgent national importance. No country can afford to neglect the enormous loss of life which is at the present time permitted through the neglect of due precautions to prevent the spread of this complaint, which probably next to small-pox is one of the most easily prevented of all the infectious diseases. The expense of legislation which has for its object the saving of upwards of 100,000 lives annually in the United Kingdom alone can hardly be expected for one moment to have any force as an argument against the undertaking. The effect of compulsory isolation in cases of scarlet fever, which was rendered possible by the establishment of the Metropolitan Asylums Board in 1867, and the building of special fever hospitals under the management of the Board, the first hospital being opened in 1869, was to reduce the average mortality from that disease per million of persons living from 1,018 between the years of 1860 and 1869 to 200 between the years of 1890 and 1899. The prevention of contagion from scarlet fever, where one contact only with an infected individual is sufficient to give rise to the illness in another person, would appear to be much more difficult to effect than the prevention of consumption, where prolonged and repeated exposure is as a rule necessary for infection. From a purely philosophical point of view it is almost amazing that human beings should allow themselves to be ousted in the struggle for existence by such a lowly vegetable parasite as the bacillus of tuberculosis.

